# Editorial: The roles of pathogens in gut-related diseases and the strategies for inhibiting their growth

**DOI:** 10.3389/fphar.2024.1513694

**Published:** 2024-12-04

**Authors:** Imran Khan, Huang Gouxin, Shafia Khan, Abid Ali

**Affiliations:** ^1^ Department of Biotechnology, Faculty of Chemical and Life Sciences, Abdul Wali Khan University Mardan, Mardan, Khyber Pakhtunkhwa, Pakistan; ^2^ Clinical Research Center, Shantou Central Hospital, Shantou, China; ^3^ Government Girls Degree College Rustam, Higher Education Department, Mardan, Khyber Pakhtunkhwa, Pakistan; ^4^ Department of Zoology, Faculty of Chemical and Life Sciences, Abdul Wali Khan University Mardan, Mardan, Khyber Pakhtunkhwa, Pakistan

**Keywords:** gut microbiota, microbiome, bacteria, pathogen, prebiotics, dysbiosis, probiotics

## Introduction

Healthy life is inconceivable without balanced microbial communities in the gut, known as the gut microbiome (GM), which play a critical role in digestion, metabolism, immune function, and overall health (Zhang et al., 2023). These commensals are sensitive to both internal and external factors that cause dysbiosis—a condition in which the diversity and composition of GM are disturbed. This imbalance negatively affects the complex relationship between the host and its microbiome (Wu et al.).

**FIGURE 1:**
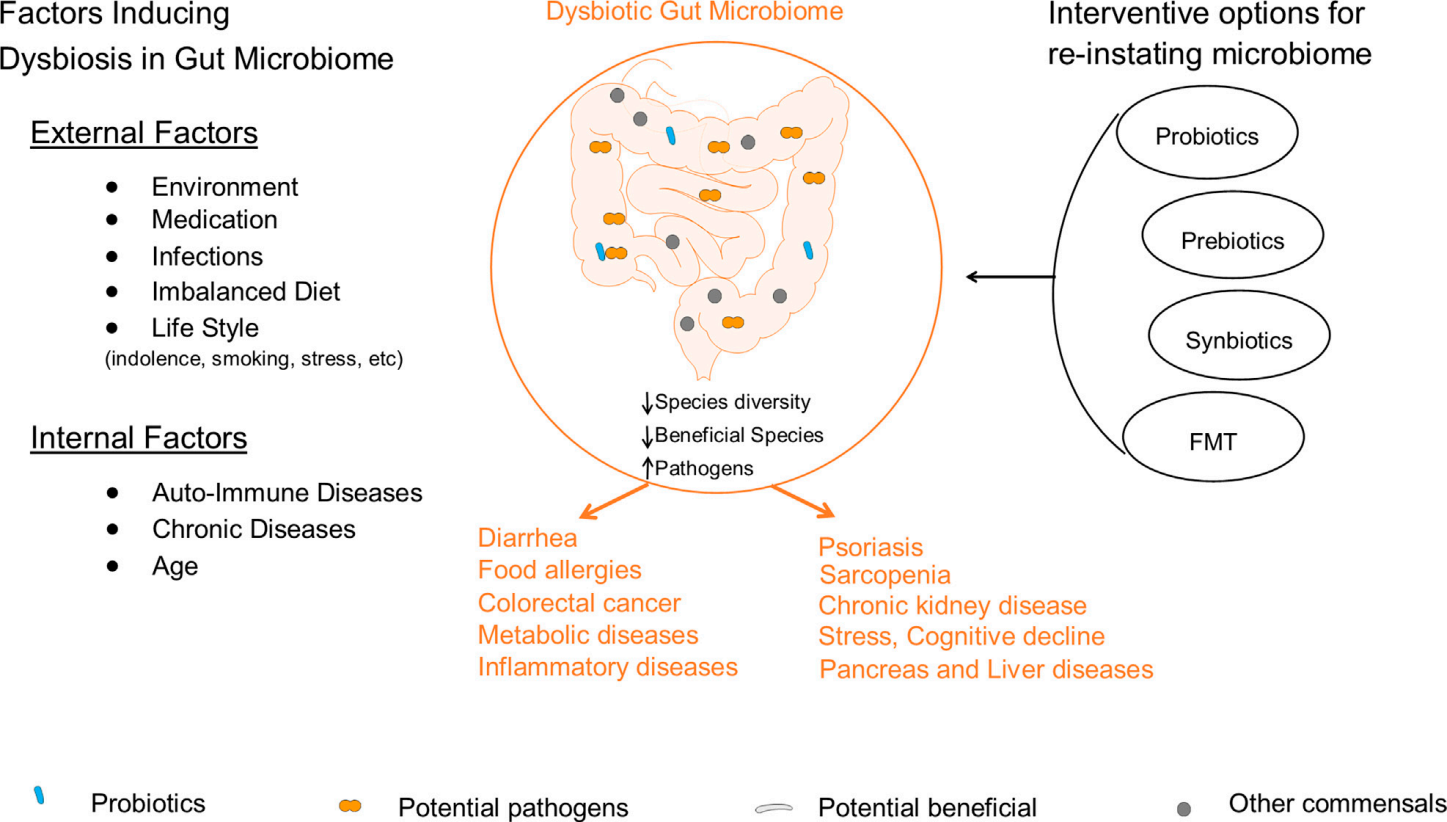
Illustration of internal and external factors that induce dysbiosis in gut microbiome and the associated consequences on health. These factors induce dysbiosis in GM by lowering the abundance of beneficial bacteria and elevating the growth of potential pathogens. The disturbed GM composition initiates/participates in the development of various diseases such as diarrhea, food allergies, cancer, metabolic diseases, stress, sarcopenia and other diseases. So far, several strategies are developed for reinstating the balance state of GM that include probiotic, prebiotics, synbiotics, and FMT interventions.

During dysbiosis, opportunistic pathogens dominate the gut ecosystem, individually or collectively, triggering serious health problems such as gastrointestinal infections, ulcers, inflammation, cancer, diarrhea, obesity, and other diseases (Wu et al.; Alam et al.). Factors that induce dysbiosis in GM include environment, imbalanced diet, chronic diseases, infection, and medication (Figure 1).

Among the medicinal treatment, antimicrobial agents induce drastic dysbiosis in the GM that trigger the emergence of opportunistic pathogens. There is growing evidence that has reported opportunistic infections supported by gut-dwelling bacteria that are commonly considered beneficial. One such bacterium is *Bacteroides fragilis* (normally a harmless gut inhabitant) that proliferates and cause infections after the antimicrobial therapies. Certain strains of this bacterium are linked to peritonitis and intra-abdominal abscesses (Jasemi et al., 2021). Similarly, the overgrowth of *Clostridium difficile* following antibiotic treatment can lead to antibiotic-associated diarrhea and, and in severe cases, can cause pseudomembranous colitis (López-Ureña et al., 2016).

The overabundance of opportunistic pathogens further worsens GM dysbiosis and cause serious complications that reach beyond the gastrointestinal tract, impacting various parts of the body. For example, *Streptococcus gallolyticus*, a gut-resident commensal, can breach a compromised intestinal barrier, enter the bloodstream, and cause infective endocarditis in individuals with damaged heart valves (Lourenc et al., 2010). Additionally, an increased prevalence of *Prevotella* species, typically gut commensals, has been associated with the onset of rheumatoid arthritis (Abdelsalam et al., 2023). Likewise, *Helicobacter pylori*, which infects more than half of the global population and, if left untreated, can lead to chronic gastritis, cap polyposis (Lu et al.), and peptic ulcer disease (Cheng et al.).

Realizing the importance of this growing enigma, we believed that the role of pathogens and opportunistic microbes in the onset, progression, and control of various diseases demand comprehensive attention. To gain deeper insight into the mechanisms underlying the pathogen-induced dysbiosis and its effects on host’ health, we initiated a Research Topic to provide a collaborative platform for researchers at the forefront of this field. In response to our call for submissions, we received 25 articles, of which only nine met publication standards. In the following section of this editorial, we provide a comprehensive summary of these manuscripts, focusing on the role of pathogens in gut microbiota dysbiosis, their impact on the host, and potential strategies for pathogen control. This section also highlights the current state of knowledge and identifies key areas for future research.

### The impact of dysbiosis extends beyond the gastrointestinal tract

Numerous studies have demonstrated that dysbiotic microbial communities disrupt the gut barrier, lead to immune imbalances, and contribute to the development of chronic metabolic diseases. Even the intake of an imbalanced diet can have profound effects on the gut microbiome. In a recent study, Lu et al. fed rats an adenine-rich diet, which resulted in reduced diversity and compositional variation of GM compared to control rats. Pathophysiological analysis of the test group revealed calcification in the kidneys and thoracic aorta. Notably, vascular calcification is a recognized risk factor for cardiovascular disease, particularly in patients with chronic kidney disease (CKD). Furthermore, an increase level of *Acinetobacter* was noticed in the blood of the test group which was positively associated with increase in calcification factors (such as BSP, FGF-23, and SOST), lipopolysaccharide, phosphorous, and genes involve in the mineral absorption pathway. The authors suggest that elevated level of *Acinetobacter* in CKD holds significant potential as both a diagnostic marker and a therapeutic target. In addition, a cluster of five bacterial species has already been proposed as non-invasive diagnostic markers for CKD (Lu et al.).

Similarly, during nonalcoholic steatohepatitis (a severe form of nonalcoholic fatty liver disease), dysbiotic gut microbiota compromise the intestinal barrier, leading to increased translocation of toxic metabolites into the circulatory system and triggering liver inflammation (Huang et al.; Zhu et al., 2022). Bacteria that have been found abundant in the gut of non-alcoholic liver diseases include Proteobacteria, Enterobacteria, *Escherichia*. Whereas, bacterial metabolites that participate in the development of non-alcoholic liver disease include amino acids, bile acids, choline and butyrate (Alam et al.).

### Fixing dysbiosis is a new target for treating disorders

Fixing disturbed GM composition has emerged as a new strategy for treatment of diseases. Interventions focused at reinstating the GM—such as the use of probiotics, prebiotics, dietary modifications, fecal microbiota transplants, and microbial-targeted therapies—have exhibited potential beneficial role in lowering the disease symptoms, improving immune responses, and uplifting the overall health.

In a recent study, Tang et al. demonstrated that balancing dysbiotic GM during psoriasis, a skin condition which is characterized by inflammation, could fix systemic inflammation and alleviate psoriasis symptoms. They successfully treated psoriasis mouse model with Shenling Baizhu Powder and concluded that fixing GM could impact lipid metabolism through reducing inflammatory mediators that conferring therapeutic abilities during psoriasis management.

Similarly, Zhang et al. fixed dysbiosis through aconite, a form of anti-diarrhea Chinese medicine. Aconite promoted the growth of bacteria belonging to *Clostridia*, *Lachnospiraceae, Muribaculaceae*, *Prevotellaceae*, and *Ruminococcus* whereas, inhibited the growth of *Escherichia*-*Shigella* and *Parasutterella*. Another study has reported disturbed microbiome composition during sarcopenia, a state of pathological decline of muscle mass and strength—associated with aging. They reported 29 genera with altered abundance in sarcopenia and proposed that *Blautia*, *Lachnospiraceae*, and *Subdoligranulum* could be developed as diagnostic markers for sarcopenia (Zhou et al.).

Another interventive strategy to restore GM composition and restrict potential pathogen is the use of herbal products (Dong et al., 2024). In a recent study, Litsea cubeba essential oil was found to positively affect gut microbial communities, leading to improved digestion, enhanced growth performance, increased antioxidant levels, and more efficient nutrient absorption (Cheng et al.). Similarly, *Artemisia argyi* essential oil has demonstrated weight management effects, which may be partly explained by its ability to modulate gut microbiota composition (Wang et al., 2022). Conversely, gut bacteria also play a role in improving the absorption and effectiveness of herbal products. A recent study utilized a biotransformation-integrated network pharmacology approach to explore how gut microbiota enhances the efficacy of Astragaloside IV in treating intracerebral hemorrhage (ICH). The researchers found that GM and liver metabolism converted Astragaloside IV into metabolites with improved bioavailability and better permeability across the blood-brain barrier, thus enhancing its therapeutic potential (Hu et al., 2023).

As medicine moves toward more personalized approaches, profiling an individual’s gut microbiome before administering herbal products becomes increasingly important. For instance, a recent study showed that podophyllotoxin, a naturally occurring aryltetralin lignan, induces neurotoxicity via the microbiota-gut-brain axis. Podophyllotoxin exposure altered the composition of gut microbiota, increasing *Escherichia*-*Shigella* populations while reducing *Lactobacillus*, which elevated pro-inflammatory factors and disrupted kynurenine metabolism in the hippocampus. These changes may contribute to cognitive dysfunction and neurotoxicity (Duan et al., 2024).

In conclusion, herbal products hold greater abilities for reinstating gut microbiome balance, which in turn can positively affect host health and alleviate disease symptoms. Herbal compounds not only stop pathogenic growth but also improve microbial diversity, improving functions such as digestion, nutrient absorption, antioxidant levels, and weight management.

## Conclusion

GM dysbiosis has been linked to the development of various illnesses that results mainly from the overgrowth of opportunistic pathogens, which modulate mucosal homeostasis and affect distant organs such as the kidneys, heart, liver, and pancreas. Various therapeutic strategies are being developed to restore GM diversity and composition for alleviating disease symptoms and improving overall health. Among these strategies, prebiotics and probiotics target the promotion of beneficial bacteria and improve resilience against pathogenic colonization. These strategies fix dysbiotic GM which consequently treat systemic diseases that are associated with dysbiosis, thus creating the possibility for a novel field in preventative and therapeutic medicine.
